# Stem cell-based bone and dental regeneration: a view of microenvironmental modulation

**DOI:** 10.1038/s41368-019-0060-3

**Published:** 2019-08-19

**Authors:** Chenxi Zheng, Ji Chen, Shiyu Liu, Yan Jin

**Affiliations:** State Key Laboratory of Military Stomatology & National Clinical Research Center for Oral Diseases & Shaanxi International Joint Research Center for Oral Diseases, Center for Tissue Engineering, School of Stomatology, The Fourth Military Medical University, Xi’an, Shaanxi China

**Keywords:** Mesenchymal stem cells, Regeneration, Stem-cell niche, Stem-cell therapies, Mechanisms of disease

## Abstract

In modern medicine, bone and dental loss and defects are common and widespread morbidities, for which regenerative therapy has shown great promise. Mesenchymal stem cells, obtained from various sources and playing an essential role in organ development and postnatal repair, have exhibited enormous potential for regenerating bone and dental tissue. Currently, mesenchymal stem cells (MSCs)-based bone and dental regeneration mainly includes two strategies: the rescue or mobilization of endogenous MSCs and the application of exogenous MSCs in cytotherapy or tissue engineering. Nevertheless, the efficacy of MSC-based regeneration is not always fulfilled, especially in diseased microenvironments. Specifically, the diseased microenvironment not only impairs the regenerative potential of resident MSCs but also controls the therapeutic efficacy of exogenous MSCs, both as donors and recipients. Accordingly, approaches targeting a diseased microenvironment have been established, including improving the diseased niche to restore endogenous MSCs, enhancing MSC resistance to a diseased microenvironment and renormalizing the microenvironment to guarantee MSC-mediated therapies. Moreover, the application of extracellular vesicles (EVs) as cell-free therapy has emerged as a promising therapeutic strategy. In this review, we summarize current knowledge regarding the tactics of MSC-based bone and dental regeneration and the decisive role of the microenvironment, emphasizing the therapeutic potential of microenvironment-targeting strategies in bone and dental regenerative medicine.

## Introduction

Bone and dental loss and defects caused by diseases and trauma have become a global concern with high incidence, which seriously affects the health and life quality of the whole population and lays a heavy financial burden on society.^1,2^ Currently, autogenous bone transplantation is the gold standard treatment for bone defects.^3,4^ For oral diseases, dental prostheses, periodontal treatment and dental implants remain the mainstream therapies.^2,5^ However, the application of autogenous bone transplantation is seriously restrained by limitations of sources, difficulty in graft harvest and morbidity of donor site.^6,7^ Moreover, present therapies for oral diseases can only improve clinical diagnostic parameters and halt disease progression but fail to regenerate lost tissue.^2,8^ Therefore, new technologies are in high demand to achieve excellent regeneration of bone and dental tissues.

Mesenchymal stem cells (MSCs), which can be isolated from different tissues and possess self-renewal and multiple differentiation potential, play an essential role in organ development and postnatal repair.^9–11^ A variety of studies, via animal models and clinical trials, have demonstrated that both endogenous and exogenous MSCs hold enormous promise in regenerative medicine for bone and tooth,^12–15^ among which bone marrow MSCs (BMMSCs) have received much attention. In addition, adipose-derived MSCs (ADMSCs)^16,17^ and dental stem cells (DSCs),^18,19^ including dental pulp stem cells (DPSCs), periodontal ligament stem cells (PDLSCs), stem cells from human exfoliated deciduous teeth (SHED), stem cells from the apical papilla (SCAP) and dental follicle cells (DFCs), have emerged as attractive cell sources for bone and dental regeneration due to their ease of accessibility and relative abundance. In addition to differentiation potential, the ability of MSCs to regulate the function of other cells and to modulate the systemic inflammatory condition via cell–cell interaction or paracrine mechanism also contributes to their therapeutic efficacy.^20,21^ Presently, there are two main strategies of MSC-based bone and dental regeneration: the rescue or mobilization of endogenous MSCs and the application of exogenous MSCs in cytotherapy or tissue engineering. Nevertheless, despite much progress, establishing safe, effective and simple stem cell-based approaches for bone and dental repair and regeneration remains a tremendous challenge,^8,14,15,22^ especially considering the adverse effects of a diseased microenvironment.^21^

In recent years, the microenvironment has been uncovered to exert enormous influence on the physical functions and pathologic changes as well as the therapeutic effects of stem cells.^23–25^ Physiologically, the niche where MSCs reside consists of a variety of tissue components, cell populations and soluble factors, which tightly regulate the behaviours of MSCs.^26–28^ Under pathological conditions, such as osteoporosis and periodontitis, both the viability and differentiation of MSCs are seriously impaired, leading to disease aggravation and impaired tissue healing.^21,29–31^ Furthermore, in cytotherapy and tissue engineering, the microenvironments of donors and recipients play a pivotal role in determining the regenerative efficacy of transplanted MSCs,^32,33^ further indicating a critical role of cell–microenvironment interactions in MSC-mediated bone and dental regeneration.

In addition to accumulating evidence identifying the prominence of the microenvironment in MSC-based regenerative therapies, solutions have been developed, such as the improvement of the microenvironment to restore endogenous MSC function, the enhancement of MSC resistance to a diseased microenvironment, and the restoration of the recipient microenvironment to benefit transplanted MSCs. Notably, after being transplanted into recipients, MSCs act as potent microenvironment modulators in both tissue engineering and cytotherapy.^34–38^ Furthermore, cell-free therapies represented by the application of MSC-derived extracellular vesicles (EVs) have become a promising alternative to whole-cell treatment.^39–41^ In this review, we overview the principles and cutting-edge progress of MSC-based tactics in bone and tooth regeneration and further highlight the involvement of the microenvironment, especially under pathological conditions. We also propose strategies to optimize MSC-based regeneration of bone and dental tissues, mainly focusing on modulating the microenvironment.

## MSCs in bone and tooth regeneration

Among the various stem cell types used for cytotherapy and tissue engineering, MSCs are currently proposed as an attractive cell source for bone and tooth regeneration due to their potential for differentiation into osteoblasts or odontoblasts, ability to modulate systematic immunity, and lack of ethical controversies.^12,14,15,22^ In addition to being classically obtained from bone marrow, MSCs can also be isolated from diverse neonatal and adult tissues, which provide more accessible sources of MSCs for bone and dental tissue regenerative therapies.^18,42,43^

### BMMSCs

BMMSCs were first discovered by Friedenstein et al.^44^ as a subpopulation of non-haematopoietic stromal cells residing in bone marrow that were able to self-renew and differentiate into multiple cell types. Since then, BMMSCs have become the most extensively studied MSCs for bone regeneration due to their intimate involvement in bone physiology and pathology.^3,45^ During adult life, bone homoeostasis maintenance depends largely on the balance between bone formation and resorption,^46,47^ which, at the cellular level, is intensely modulated by BMMSCs via differentiating into osteoblasts and regulating osteoclasts’ activities.^10,48^ Pathologically, BMMSC dysfunction has been revealed to be a critical cellular mechanism underlying various bone disorders, especially osteoporosis.^21,30,31^ More importantly, BMMSCs act as potent microenvironmental modulators that exert enormous anti-inflammatory effects after systemic transplantation, which benefit diverse tissues/organs, including bone.^35,36,41^ Accordingly, a variety of studies have shown promising therapeutic potential of BMMSCs in osteopenia and bone defects via cytotherapy or tissue engineering construction.^33,41,49–52^

### ADMSCs

Since their discovery by Zuk et al.^53^ in 2001, ADMSCs have been increasingly demonstrated to hold great promises in regenerative medicine. Similar to BMMSCs, ADMSCs display steady growth kinetics in vitro and are able to differentiate into various cell types, including osteocytes, chondrocytes and adipocytes.^16,17^ In addition, with a prevalence of lipoaspirates and less morbidity to the host during procurement, ADMSCs are, to some extent, more advantageous than BMMSCs due to easy accessibility and abundant supply.^16,17,54^ Furthermore, compared to BMMSCs that are prone to pathological factors of bone, ADMSCs demonstrate functional maintenance in various bone pathological conditions, as demonstrated by the preservation of cell viability, differentiation capacity,^29,55–58^ and more importantly, therapeutic efficacy.^59–61^ Recent studies have revealed the efficacy of ADMSCs in repairing critical-sized bone defects, improving osteopenia and constructing engineered bone grafts and have proposed these cells as an excellent alternative to BMMSCs.^59–63^

### DSCs

**Fig. 1:**
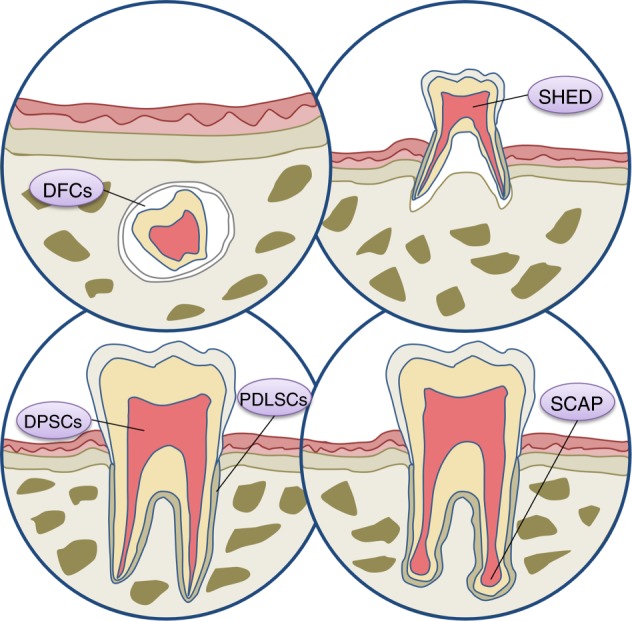
Stem cell populations derived from different dental tissues/regions that constitute appealing MSC sources for bone and dental regeneration. DFCs, stem cells from dental follicle; SHED, stem cells from human exfoliated deciduous teeth; DPSCs, stem cells from dental pulp; PDLSCs, stem cells from PDL; SCAP, stem cells from the apical papilla

A variety of stem cell populations have been obtained from diverse parts of the tooth with a common neural crest origin and generic MSC-like properties, including the expression of specific surface markers and potential to differentiate into mesenchymal cell lineages^18,19^ (Fig. 1). Remarkably, DSCs exhibit many advantages, such as easy accessibility, abundant source and less inconvenience to donors,^14,54^ which constitutes an appealing source of autologous MSCs, especially for the regeneration of pulp tissue and periodontal ligament (PDL) and the production of partial or whole tooth structures for biological implant construction.^14,18,19,54^

#### DPSCs

Derived from pulp that is entrapped within the pulp chamber and possessing good reparative and regenerative abilities, DPSCs are the first DSCs to be discovered by Gronthos et al.^64^ in 2000 with self-renewal capability and multilineage differentiation potential. DPSCs are essential for postnatal tooth homoeostasis and repair due to their ability to replenish odontoblasts during the restoration of dentin.^65–67^ In addition, since DPSCs are of neural origin, these cells are able to differentiate into functionally active neurons and glial cells upon proper environmental stimulation.^68,69^ In addition, DPSCs demonstrate a distinguishing capacity to secrete neurotrophic factors that contribute to neuroprotection and neurite outgrowth.^68,69^ More importantly, recent studies have revealed that DPSCs reside in a neurovascular bundle niche.^70^ Intriguingly, in accord with their perivascular residence, DPSCs possess strong angiogenic ability,^69^ as shown by their abilities to secrete an array of angiogenic regulatory factors and to generate capillary-like structures under particular environmental conditions. Taken together, the MSC-like and neurovascular properties of DPSCs, owing to neural crest or glial origins during development and subsequent fostering by the neurovascular niche during growth, makes these cells an optimal population for bone and tooth regeneration.^71–73^

#### PDLSCs

PDL is a fibrous connective tissue located between the cementum of the root and the alveolar bone socket wall, which plays an important role in supporting the teeth via anchoring the tooth to alveolar bone.^54^ PDLSCs, a stem cell subpopulation first discovered by Shi et al.^74^ from PDL, are responsible for the physical maintenance and regeneration of periodontal tissue structure and function. Upon in vitro culture, these cells possess a clonogenic nature, express a variety of cementoblastic/osteoblastic markers and are able to form mineralized nodules. Moreover, after in vivo transplantation, PDLSCs are able to form cementum- and PDL-like structures.^74^ Regarding periodontal defects, locally transplanted PDLSCs migrated into the PDL section and successfully repaired defects, implying the potential of PDLSCs in periodontal tissue regeneration.^75,76^ In addition, an optimal protocol has been established with regard to the extraction, expansion and characterization of human PDLSCs, which are usually obtained from extracted orthodontic teeth or normal impacted third molars.^18^ Furthermore, PDLSCs can also be obtained from residual PDL on retained deciduous teeth^77^ or cryopreserved human PDL,^78^ which expands the sources of PDLSCs.

#### SHED

In 2003, Miura et al.^79^ isolated a population of MSCs from the pulp tissue of the crown of exfoliated deciduous teeth differing from DPSCs and named them SHED. After in vivo implantation, SHEDs are capable of forming dentin-like structures, indicating their potential in pulp regeneration.^79–81^ In addition, compared with DPSCs, SHEDs exhibit higher proliferative activity, odontogenic and osteogenic differentiation potential, and osteo-inductive ability.^82,83^ In addition, SHEDs are able to differentiate into neuronal and glial cells when cultured within neurogenic inductive media.^79,84^ Furthermore, SHEDs can be harvested via a relatively easy approach and maintain their regenerative potential after cryopreservation for cell banking, as demonstrated by the maintenance of surface antigens and differentiation properties after 2 years of cryopreservation.^85^ Remarkably, our group demonstrated that the implantation of SHED regenerated three-dimensional whole dental pulp accompanied by blood vessels and nerves in both animal models and patients with tooth trauma,^71^ further supporting SHED as an attractive cell source for bone and tooth regeneration.

#### SCAP

Since apical papilla tissue only exists during root development before the tooth erupts into the oral cavity, SCAP is a unique population of DSCs located at the tips of growing tooth roots.^86–88^ These cells possess MSC properties, including clonogenicity and the ability to differentiate into odontoblasts/osteoblasts in vitro.^86,88^ In addition, SCAP demonstrated higher proliferation rates and stronger mineralization ability in vitro than DPSCs.^89^ Indeed, SCAP are able to regenerate a typical cementum/PDL-like complex in vivo, further indicating their potential for bone and dental tissue regeneration.^90^ In addition, SCAP have been reported to form a root/periodontal complex after co-transplantation with PDLSCs into tooth sockets of mini pigs.^86,88^ Moreover, cells derived from inflamed periapical tissue also exhibit typical MSC characteristics with highly osteogenic capacity in vitro and in vivo,^91^ suggesting the maintenance of stemness. Considering that roots develop postnatally and that the root apical papilla is clinically available from extracted wisdom teeth, SCAP can provide a source of MSCs that are isolated in the process of development and possess embryonic-like properties.^14,54^

#### DFCs

As a loose connective tissue derived from ectomesenchyme, the dental follicle surrounds the enamel and the dental papilla of the developing tooth germ before tooth eruption, which contains progenitors for osteoblasts, cementoblasts and PDL.^92,93^ Specifically, DFCs form the PDL via differentiation into PDL fibroblasts that secrete collagen and interrelate with fibres between the adjacent bone and cementum. DFCs are able to differentiate into cementoblasts under in vitro culture^94,95^ and to generate cementum when implanted in vivo.^93^ In addition, DFCs generated PDL-like tissue after in vivo transplantation^96^ and produced periodontal tissues via epithelial-mesenchymal interaction.^97^ Notably, DFCs maintained MSC features in culture after more than 30 passages and formed single or complex tissues in the periodontium.^98^ Collectively, DFCs are a promising MSC source for regenerative medicine.^14,54^ Furthermore, similar to SCAP, DFCs are a group of cells derived from developing tissue, thus possessing higher plasticity than other DSCs.

## Stem cell-based regenerative strategies for bone and dental tissue

### Regeneration with endogenous stem cells

**Fig. 2:**
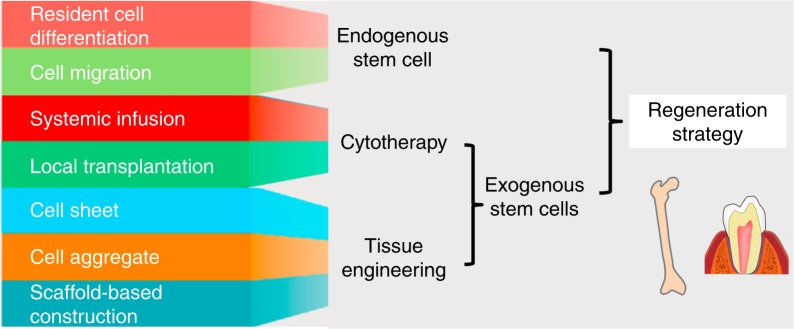
Stem cell-based regenerative strategies for bone and dental tissue. Based on the cellular sources, stem cell-based bone and dental regeneration mainly includes two categories: endogenous stem cell- and exogenous stem cell-mediated regeneration strategies. Both resident and migratory endogenous stem cells have shown great potential in healing of bone and dental loss and defects. Alternatively, the application of exogenous stem cells is promising for regenerating bone and dental tissue through either cytotherapy via systemic/locoregional infusion or tissue engineering, including cell sheet, cell aggregate and scaffold-based tissue construction

Despite the enormous efforts devoted to exogenous MSC transplantation for tissue regeneration, an alternative therapeutic strategy is to take advantage of endogenous MSCs, which reside within specific tissues and are able to self-renew and produce specific cell types^99–101^ (Fig. 2). Compared with exogenous MSC transplantation, tissue regeneration mediated by endogenous MSCs is less expensive and labour intensive and avoids surgical injury and rejection risk.^100,101^ Nevertheless, in mammals, the regenerative capabilities of endogenous MSCs progressively decline during postnatal development, leading to limited innate repairing capacity.^101^ Moreover, under pathological conditions, such as osteoporosis and periodontitis, the function of endogenous MSCs is severely impaired, as characterized by decreased proliferation and osteogenic differentiation capabilities.^21,30,31,102^ Accordingly, pharmacological approaches have been developed to rescue MSC deficiency in bone and oral tissue loss.

Mechanistically, studies have revealed a variety of molecular mechanisms underlying gene expression regulation in bone that forms a complex signalling network, including mammalian target of rapamycin signalling,^103,104^ Notch signalling,^41^ nuclear transcription factor-kappaB (NF-KB) signalling^105^ and Wnt signalling,^106^ which suggested multiple intervention targets in restoring MSC functions. Accordingly, a variety of agents targeting the above signalling pathways have been demonstrated to be effective in rescuing endogenous MSC impairment and promoting bone and dental regeneration, such as rapamycin,^103,104^ DAPT,^41^ PDTC,^105^ and dickkopf-1.^106^ In addition, antioxidants, including NAC,^107–109^ resveratrol,^110,111^ and melatonin,^112^ which protect MSCs from oxidative damage, have been successfully used to restore MSC function and improve osteopenia and periodontitis. Furthermore, approaches targeting epigenetic regulation mechanisms, such as histone modification regulators DZNep,^113^ pargyline^114^ and KDM5A,^115^ and microRNA expression regulation,^116–119^ have exerted therapeutic efficacy on osteoporosis, fracture healing and oral tissue regeneration. In addition to modulating in situ MSCs, the mobilization of endogenous MSCs from other sites of the body to an injury site to replenish deficient resident MSCs also contributes to tissue regeneration.^120–122^ In this regard, LLP2A-Ale, which acts as a migration stimulator, has been used to accelerate bone formation in oestrogen deficiency-induced osteoporosis via directing MSCs to bone formation surfaces.^123^ Moreover, the combination of stromal cell-derived factor-1 with a protein-releasing scaffold promoted chemotaxis-induced MSC homing, leading to the repair of bone^124^ and periodontal^125^ defects. Scaffold-releasing agents to enhance endogenous MSC functions also include transforming growth factor (TGF)-β3^126^ and fibroblast growth factor-2,^127^ which benefit bone and tooth regeneration. More importantly, the modulation of the specific microenvironment where stem cells reside in vivo is an effective way to regulate endogenous MSC behaviour, which will be discussed in detail in the following sections.

### Regeneration with exogenous stem cells

Within recent decades, the transplantation of exogenous MSCs through different routes has been widely explored in bone and dental regenerative medicine (Fig. 2). A promising strategy is the systemic application via primarily intravenous infusion and intraperitoneal delivery, which exerts therapeutic effects on various disorders, including osteoporosis, bone fracture, osteoarthritis and jaw osteonecrosis. Mechanistically, through homing to recipient bone marrow or fracture sites, infused MSCs promote osteogenesis by differentiating into osteoblasts, inducing endogenous osteoblastogenesis and modulating osteoclast-mediated bone resorption.^49,128–130^ In this regard, genetic or pharmacological approaches that enhance the homing of MSCs could strengthen their therapeutic efficacy on bone loss and defects.^131–133^ More importantly, systemically transplanted MSCs exhibit enormous potential to modulate systemic immunity, especially by suppressing inflammation to restore the recipient microenvironment, which also contributes to their therapeutic effects.^36,37,134–137^

As alternatives to single cell suspension injection, cell sheets and aggregates provide novel strategies for cell delivery without scaffolds, enabling stable engraftment and long-term viability of transplanted cells.^138–142^ With the application of temperature-responsive culture dishes, cultured cells could be non-invasively harvested as whole sheets together with their deposited extracellular matrix (ECM), which can be directly transplanted to host tissues in vivo or used to produce tissue constructs in vitro.^138,140^ In addition, transplanted cell sheets spatiotemporally release growth factors and modulatory cytokines, further contributing to tissue regeneration.^54,142^ To date, BMMSC-based cell sheets have been successfully used to promote bone regeneration in defects^52,143^ and strengthen implant bone bonding.^144^ In addition, PDLSC sheets have been demonstrated to promote periodontal regeneration in various animal species.^145–149^ After in vivo grafting of monolayered or layered PDLSC sheets, the cement/PDL complex was observed along with well-oriented collagen fibres, leading to the repair of periodontal defect. More importantly, clinical trials conducted on periodontitis patients further indicate the periodontal regeneration potential of PDLSC sheets.^150,151^

Moreover, cell aggregates produced via micro-mass pellet culture of groups of cells under certain conditions have emerged as an attractive strategy in regeneration medicine due to structural and functional similarity to native tissue.^54,141,142^ Compared with single cells or cell sheets, cell aggregates contain more ECM and thus may possess more biological and inductive activities.^139,142^ BMMSC-based aggregates could promote bone formation in a metaphyseal defect model of ovariectomized (OVX) rats,^50^ and PDLSC aggregates showed promise in healing periodontal defects.^152,153^ In addition, the application of composite cell aggregates with PDLSCs and jaw-derived BMMSCs resulted in the generation of functional PDL-like tissue both ectopically in nude mice and orthotopically in minipig.^154^ Notably, a clinical trial conducted by our group revealed that SHED aggregates could regenerate complete pulp tissues in patients with pulp necrosis, which were equipped with blood vessels and nerves.^71^ More importantly, after SHED implantation, the recipient immature permanent teeth showed increased root length and decreased apical foramina width, suggesting that the regenerated pulp acted as normal pulp with the ability to maintain continued root development.^71^

The combination of cells with scaffolds and bioactive factors, which is a classical tissue engineering strategy, is also promising for bone and dental regeneration.^3,4,6,7,14^ Typically, bone scaffolds are made of biomaterials, including bioactive ceramics, biodegradable metals, biodegradable polymers and calcined bone, which serve as 3D structures that lead to cell migration, proliferation and differentiation.^22^ Moreover, pharmacological modification of scaffolds enables the release of biological molecules, such as angiogenic factors and osteogenic factors, thus modulating the activity and function of seeded cells and endogenous cells.^7,21,22^ The transplantation of BMMSC sheets combined with polycaprolactone–calcium phosphate (PCL–CaP) scaffolds in vivo resulted in the formation of neo cortical and well-vascularised cancellous bone in rats.^155^ In addition, the application of calcined bovine bone coated with BMMSC sheets repaired critical size bone defects in osteoporotic rats.^52^ Moreover, PDLSC sheets combined with β-tricalcium phosphate (β-TCP)/collagen scaffolds or PCL–CaP scaffolds promoted periodontal regeneration with newly formed cementum and well-oriented PDL fibres.^146,156^ Based on current information available, an exogenous MSC-based regenerative strategy constitutes a promising approach for healing bone and dental loss and defects.

## Microenvironmental impact on stem cells in bone and dental regeneration

**Fig. 3:**
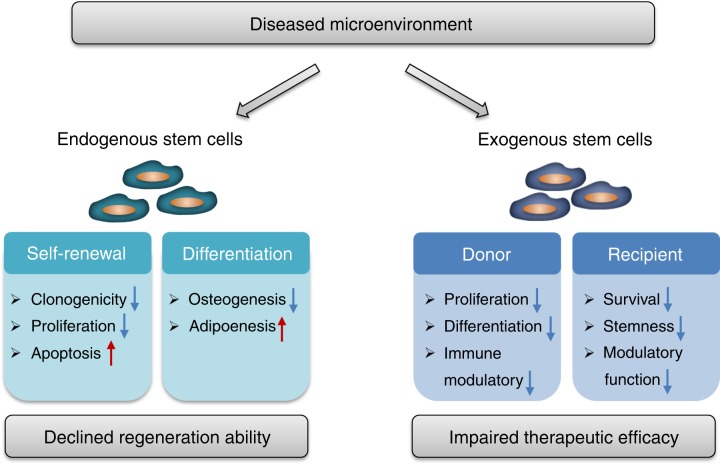
Microenvironmental impact on stem cells in bone and dental regeneration. Under pathologic microenvironmental conditions, the survival and functions of endogenous stem cells are impaired, as shown by declined self-renewal ability and disturbed differentiation potential, leading to development of bone diseases and declined regeneration ability. On the other hand, the therapeutic efficacy of transplanted exogenous MSCs are compromised by the diseased microenvironments of the donors where they are harvested and of the recipients where they are applied due to the impairment of stemness and immune modulatory function

The microenvironment, which acts as “soil”, has been increasingly recognized to exert tremendous effects on MSCs, the “seeds” in organs, especially under pathological conditions and in cell therapies (Fig. 3). As discussed above, endogenous MSCs reside in a dynamic and complex niche consisting of neighbouring cells, ECM and plentiful neurovascular bundles, which tightly control MSC behaviours.^27,70^ In addition, resident MSCs are modulated by the circulatory microenvironment through hormones, metabolites, inflammatory cytokines and other soluble factors.^31^ Notably, microenvironmental alterations play a pivotal role in the initiation and deterioration of skeletal and dental diseases.^157^ Under pathologic microenvironmental conditions, the survival and functions of endogenous MSCs are impaired, leading to declined regeneration capacity and extensive bone loss.^31^ On the other hand, the regenerative ability of transplanted exogenous MSCs is strongly influenced by the donor microenvironmental condition where they are harvested and the recipient microenvironmental niche where they home.^21^ Consequently, achieving therapeutic efficacy in diseased microenvironments with host comorbidities constitutes a major challenge to MSC-based regeneration at the present time. Further understanding of the impacts exerted by the diseased microenvironment on MSCs and the underlying mechanism would promote the optimization of strategies that regenerate bone and oral tissue via targeting endogenous MSCs and transplanting exogenous MSCs.

### Impairment of endogenous stem cells by pathogenic microenvironment

As important systemic factors, steroid hormones, such as sex hormones (oestradiol and testosterone) and glucocorticoids, are responsible for modulating bone development and postnatal remodelling. Pathologically, hormonal disorders, particularly the dramatic decline in sex hormones in aged people and postmenopausal women, cause an imbalance between osteoblastogenesis and osteoclastogenesis, leading to the loss of bone mass and strength. Studies have demonstrated that a deficiency in oestrogen and androgen impairs the proliferation and osteogenic differentiation of BMMSCs, as shown by diminished clonogenic assay, mineral nodule formation and osteogenic marker expression.^158–160^ In addition, the ability of MSCs to generate new bone ectopically is also compromised by ovariectomy.^37^ In addition, the differentiation potential of BMMSCs is shifted towards adipocytes, leading to bone marrow fat accumulation and bone loss.^30,161^ Mechanistically, in addition to indirect effects via immune reactions, oestrogen could directly maintain MSC functional homoeostasis through binding to its receptors.^162,163^ Moreover, oestrogen deficiency causes a dramatic accumulation of reactive oxygen species (ROS), which act as an important mediator in BMMSC specification impairment^107,160,162,163^ and induce the apoptosis of BMMSCs.^164^ Recently, epigenetic mechanisms, including histone methylation,^113^ histone acetylation^165^ and microRNAs,^119,160,161,166^ have been revealed to also participate in oestrogen deficiency-mediated BMMSC impairment. In glucocorticoid-induced osteoporosis, the most prevalent form of secondary osteoporosis, excessive use of glucocorticoid also suppresses BMMSC proliferation and impairs the osteogenic potential of BMMSCs, resulting in bone loss.^167^

Another pronounced microenvironmental factor influencing bone homoeostasis is organismal metabolism. At the cellular level, the energy metabolic profiles, mainly referring to the states of glycolysis and OXPHOS, are highly influential on the fate of stem cells during development and regeneration.^167–169^ Notably, BMMSCs rely on glycolysis to maintain stemness while requiring glucose uptake and transformation into OXPHOS-privileged status when undergoing osteogenic differentiation.^170,171^ Nevertheless, excessive exposure to glucose causes BMMSC dysfunction with increased senescent phenotypes,^172,173^ which is associated with the adverse impacts of hyperglycaemia on BMMSCs, as shown in diabetic patients. Diabetes, a common metabolic disease characterized by high blood sugar, impairs multiple organ systems, including bone and oral tissue, leading to an increased risk of osteoporosis, fractures and periodontitis.^174^ The overall bone turnover rate is downregulated under hyperglycaemic conditions, especially bone formation parameters, as shown by reduced osteoblast numbers and decreased osteoid quantities. A pivotal mechanism underlying the diabetic osteogenesis decline is the accumulation of advanced glycation end products, which impair viability and osteogenic differentiation of BMMSCs by directly activating the receptor.^30^ In addition, oxidative stress also contributes to BMMSC dysfunction caused by hyperglycaemia.^175^ In addition, hyperglycaemia increases mitochondrial accumulation of P53, which recruits Bax and activates molecular events of apoptosis, leading to a decline of the number of BMMSCs.^176^ Furthermore, diabetes is an established risk factor for periodontal diseases with increased prevalence and severity.^177^ The multiple differentiation potential of PDLSCs derived from periodontitis patients with diabetes mellitus was dramatically impaired,^178^ which may involve activation of the receptor for AGEs^179^ and oxidative stress.^180^

Despite the essential role of inflammation in bone fracture healing, a pro-inflammatory microenvironment is a pivotal pathogenetic mechanism underlying various osteopenic disorders.^181^ Indeed, the induction of bone loss by pro-inflammatory cytokines occurs not only in inflammatory and autoimmune diseases but also in oestrogen-deficient, hyperglycaemic and aging conditions as key secondary detrimental factors.^104,181,182^ In particular, inflammatory cytokines, including tumour necrosis factor-α (TNF-α) and interferon-γ (IFN-γ), synergistically induce osteogenic differentiation dysfunction of BMMSCs,^166,182^ which involves signalling pathways and/or epigenetic modulations. In addition, the inflammatory microenvironment causes overproduction of ROS due to the compromised function of the mitochondrial electron transport chain and antioxidant system, which also contributes to the impairment of BMMSC lineage allocation. Moreover, high levels of TNF-α have been demonstrated to induce apoptosis and inhibit the proliferation of MSCs, leading to augmented bone loss and delayed fracture healing.^32,160^ Furthermore, the inflammatory microenvironment acts as a key contributor to the decreased osteogenic differentiation of PDLSCs in periodontitis by intervening with the expression of signalling molecules^106^ and posttranscription modulation.^183–185^ Intriguingly, the chronic inflammation of periodontitis impairs endoplasmic reticulum function and induces endoplasmic reticulum stress, leading to defective osteogenic differentiation of PDLSCs.^102^ Taken together, the diseased microenvironment plays an important role in MSC dysfunction, which provides pivotal targets for treating bone and oral diseases via rescuing endogenous MSCs.

### Influence of donor microenvironment on harvested stem cells

Different MSCs reside in different niches, which not only contain tissue-specific structures and components but also possess distinct properties due to contact with the systemic circulation and the external environment. In particular, oral tissues that are exposed directly to the outside environment are more prone to be affected by the surroundings. Despite the common characteristics, studies have found that MSCs derived from different sources are, to some extent, functionally distinct, leading to therapeutic discrepancies in cytotherapy. For example, compared to BMMSCs, DPSCs and SHED that are derived from neural origins are capable of differentiating into functionally active neurons and glial cells with proper environmental cues and can secrete neurotrophic factors for neuroprotection and neurite outgrowth.^68,69^ In this regard, DPSCs and SHED may be more advantageous for neural regeneration. In addition, SHEDs showed stronger proliferative ability than DPSCs, with higher expression of genes involved in cell proliferation and ECM formation.^82,83^ In addition, PDLSCs possess higher differentiation potential than gingival MSCs (GMSCs),^186^ while SCAP demonstrate high proliferative activity than DPSCs and PDLSCs.^89^ Molecularly, the discrepancy of different tissue-derived MSCs involves signalling pathways and epigenetic mechanisms. During osteogenic differentiation, Wnt signalling is more essential for BMMSCs, while the BMP pathway plays a more important role in ADMSCs, which leads to different responses of BMMSCs and ADSMCs to microRNA regulation.^119^ Further deciphering the distinct transcriptional and posttranscriptional regulation of different MSCs would help clarify the specificity of tissue-specific MSCs, thus promoting the development of MSC-based cell therapy.

As discussed above, a diseased microenvironment induces endogenous MSC dysfunction, which is also reflected in their therapeutic efficacy when used as exogenously transplanted MSCs. Accordingly, the influence of the donor microenvironment on MSCs is a critical issue to be considered when applying cytotherapy, especially autologous cell transplantation. Intriguingly, recent studies have revealed that under pathological conditions, different tissue-derived MSCs showed different phenotypes, which may result in discrepancies in their therapeutic efficacy. Although PDLSCs are the first choice of MSCs for periodontal regeneration, these cells are more easily impaired by the inflammatory microenvironment than GMSCs in terms of both in vitro osteogenic differentiation ability and in vivo bone formation.^186^ Notably, ADMSCs are more functionally stable than BMMSCs, which are prone to bone pathogenesis and display impaired regenerative potential. Unlike BMMSCs, ADMSCs derived from aged and oestrogen-deficient osteoporotic donors preserve cell viability and osteogenic differentiation potential in vitro and, more importantly, maintain regenerative abilities for bone loss and defects when transplanted in vivo.^55–60,187,188^ Furthermore, ADMSCs derived from OVX mice have been demonstrated to preserve anti-inflammatory capacity and alleviate bone loss in OVX recipients via systemic delivery, which implies that ADMSCs are a promising source for osteoporotic cytotherapy with resistance to a diseased microenvironment.^59^ Mechanistically, ADMSCs derived from osteoporotic donors maintain stemness, energy metabolism status and antioxidative defence system as well as preserved expression levels of immunomodulatory genes.^59^ Considering the difference between distinct MSC types in cellular properties and resistance to diseased microenvironments, evaluation and selection of MSC sources is therefore beneficial for fulfilling the therapeutic efficacy of MSC-based bone and dental regeneration.

### Impact of recipient microenvironment on transplanted stem cells

In recent years, studies have gradually recognized the profound effects of recipient microenvironmental status on the therapeutic efficacy of MSCs.^21,59^ Since it is easier for MSCs to generate new bone in immunocompromised mice, studies have revealed that the recipient pro-inflammatory T cells suppress the regenerative potential of MSCs through the synergistic effects of IFN-γ and TNF-α, which inhibit MSC osteogenesis and induce MSC apoptosis.^34^ Correspondingly, the systemic injection of T-regulatory cells (Tregs), which inhibits recipient immune responses and inflammation in immunocompetent mice, could significantly promote the repair of calvarial defects by locoregionally transplanted MSCs.^34^ Moreover, the modulation of transplanted MSC performance by the recipient inflammatory microenvironment also occurs in the cytotherapy of systemically infused MSCs, although with more complicating effects. The immunosuppressive function of MSCs in vivo requires the presence of recipient IFN-γ together with other pro-inflammatory cytokines.^35^ Recently, researchers have revealed that recipient cytotoxic cells are essential for the initiation of MSC-induced immunosuppression.^189^ Nevertheless, under excessive inflammation conditions, such as the arthritic milieu, the immunomodulatory ability of MSCs is abrogated, leading to therapeutic failure.^190^ Accordingly, it is likely that a balanced recipient inflammatory microenvironment is pivotal to elicit MSC-mediated immunomodulation and bone healing, which is the case for osteoporotic models such as ovariectomy and systemic lupus erythaematosus (SLE).^37,136^

Considering that osteoporotic remedy is not always achieved even under an inflammatory microenvironment, it is possible that other microenvironmental factors also control the therapeutic effects of MSCs on bone and dental disorders. Given that diabetes is a high risk factor for bone and dental diseases and that hyperglycaemia severely impairs the function of endogenous MSCs, our group further elucidated the influence of the recipient glycaemic microenvironment on cytotherapy mediated by transplanted MSCs. We found that recipient diabetes hinders the therapeutic efficacy of systemically injected MSCs on osteoporosis, which indicates that the metabolic status of the recipient microenvironments also constitutes a critical factor determining the efficacy of MSC-based therapies.^33^ Accordingly, the injection of insulin to restore recipient glucose homoeostasis before the infusion of MSCs could recover the therapeutic effects of MSCs to treat diabetic osteopenia.^33^ Molecularly, adenosine monophosphate-activated protein kinase signalling is involved in the impairment of MSC-mediated immunomodulation by recipient hyperglycaemia.^33^ Taken together, the recipient microenvironment plays a pivotal role in controlling MSC-mediated regenerative therapy, which needs to be further elucidated to promote the development of broadly applicable strategies for bone and dental tissue regeneration.

## Strategies to optimize stem cell-based bone and dental regeneration via targeting the microenvironment

**Fig. 4:**
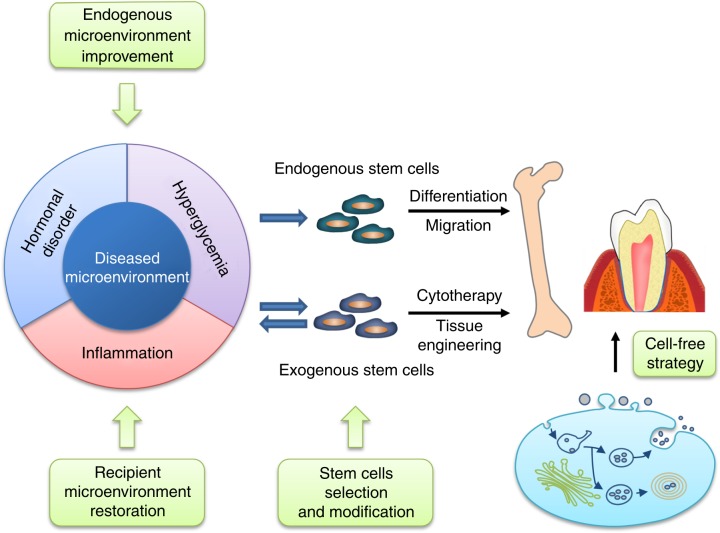
Stem cell-based bone and dental regeneration: a view of microenvironmental modulation. Diseased microenvironments, such as hormonal disorders, hyperglycaemia and inflammation, severely impair the therapeutic efficacy of both endogenous and exogenous stem cell-based regeneration of bone and dental tissue. Accordingly, microenvironmental therapeutics, including improvement of the endogenous niche, selection and modification of donor stem cells, and restoration of recipient microenvironment, have shown great promises in optimizing stem cell-based bone and dental regeneration. Furthermore, extracellular vesicles are emerging as promising cell-free strategies for bone and dental regeneration

Despite the enormous advancement achieved in recent years, stem cell-based bone and tooth regeneration are still faced with many challenges, especially low and uncertain efficacy.^8,14,15,22^ Microenvironments play a critical role in controlling both endogenous and exogenous MSC-mediated healing of bone and dental loss and defects, which underlies the failure of MSC-based therapies in diseased microenvironments.^21^ Accordingly, we envisage that strategies targeting the microenvironment will greatly promote the fulfilment of MSC therapeutic potential (Fig. 4). On the one hand, approaches aimed at correcting the diseased microenvironment could facilitate endogenous MSC-based regeneration.^24,101^ On the other hand, enhancing the resistance of MSCs to diseased microenvironment and/or pre-normalizing recipient microenvironment would enhance the therapeutic efficacy of transplanted exogenous MSCs.^21^ In addition, considering that scaffolds act as niches for seeded cells, modification of biomaterial-based scaffolds to recreate the specific and instructive microenvironment is beneficial for MSC-based tissue engineering.^6^

### Improvement of the microenvironment to restore endogenous stem cell function

Considering the aetiological role of diseased microenvironment-induced MSC dysfunction in bone and dental disorders, a promising strategy to facilitate endogenous MSC-based bone and tooth repair is to manipulate the stem cell microenvironment, which is less expensive and labour intensive and avoids surgical injury and rejection risk. Recently, tantalizing evidence has emerged that therapeutics via microenvironmental interventions could potently restore normal host conditions that rejuvenate endogenous MSCs for bone and dental healing. The application of hormonal replacement therapy for postmenopausal women with oestrogen or a combination of oestrogen and progestogen is protective against osteoporosis,^191^ which is in part due to the restoration of MSC functions. Specifically, the osteogenic potential of MSCs derived from osteoporotic patients can be promoted by oestrogen treatment but not by testosterone, as shown by increased calcium deposition and osteogenic gene expression,^162^ indicating a critical role for oestrogen in maintaining MSC functions. In addition to improving MSC-mediated osteogenesis, supplementation with oestrogen also enhances MSC-induced apoptosis of osteoclasts by preserving Fas ligand (Fasl) expression.^192^ Further studies are needed to establish medication usage to mimic the physical pattern of hormone secretion.^191^

Recently, caloric restriction that lowers glucose metabolism and nutrient intake has been increasingly recognized as a rejuvenative intervention in many organisms to rescue functional decline in various human organs.^193^ Notably, life-long caloric restriction has been demonstrated to prevent age-induced bone loss, while short-term caloric restriction exerted no effects.^194,195^ For MSCs, the downregulation of glucose levels in the in vitro culture microenvironment dramatically protected MSCs from replicative senescence after serial passages with strengthened proliferation and osteogenic potential.^172^ In addition, metabolic control of hyperglycaemia via the long-term infusion of insulin preserved bone mineral density and lowered fracture risk in diabetic patients.^196,197^ Moreover, pharmacological intervention by metabolic regulators has been reported to restore impairment of BMMSC-mediated osteoblastogenesis and alleviate bone disorders. For example, treatment with resveratrol via oral feeding or intraperitoneal injection alleviated the in vivo skeletal senescent phenotype with improvement of resident BMMSCs osteogenic potential.^110,198^ In addition, the administration of resveratrol restored ligature/lipopolysaccharide-induced alveolar bone loss, with inhibition of inflammation and oxidative stress.^111^ Moreover, the administration of metformin in drinking water or through local injection promoted BMMSC osteogenic differentiation, resulting in facilitation of bone lesion regeneration in diabetic rats^199^ and prevention of bone ageing.^200^

The above findings have highlighted the prevalence of inflammation in bone and dental disorders and the impairment of MSCs by the pro-inflammatory microenvironment. Accordingly, anti-inflammation therapy constitutes an important approach for bone and dental regeneration via restoring endogenous MSC functions. Indeed, genetic deletion of TNF-α exerts protective effects on bone mass under diseased conditions.^201^ At the cellular level, the impaired osteogenic differentiation ability of BMMSCs derived from oestrogen-deficient mice was rescued by treatment with neutralizing antibodies that deplete either TNF-α or IFN-γ.^182^ Further in vivo infusion of these antibodies also rescued resident BMMSC dysfunction and alleviated OVX-induced osteoporosis, with enhanced effects when used together.^182^ Notably, the application of anti-TNF-α at earlier and later stages of oestrogen deficiency-induced osteoporosis exerted preventive and curative effects on bone loss, respectively.^166^ Pharmacologically, anti-inflammatory drugs have been demonstrated to improve bone and dental diseases via rescuing resident MSC homoeostasis.^183,202^ Systemic administration of aspirin via oral feeding downregulated the systemic concentration of TNF-α and IFN-γ, thus restoring MSC functions and abrogating oestrogen-deficient-induced bone loss.^182,202^ Furthermore, aspirin treatment restored deficient osteogenic differentiation of PDSLCs under an inflammatory microenvironment, which upon in vivo injection, rescued periodontitis.^183^ Clinical translation of these microenvironmental therapies will help optimize endogenous MSC-based bone and dental regeneration.

### Cell source evaluation and cellular modification to obtain feasible stem cells

As discussed above, different sources of MSCs display distinct resistance to diseased microenvironments, suggesting that the selection of cell sources is essential for optimizing regenerative therapies. To date, BMMSCs are the most popular candidates in bone regeneration therapy both via cytotherapy and tissue engineering.^3^ However, BMMSC functions are prone to a diseased skeletal microenvironment, leading to therapeutic efficacy impairment of autologous cells.^60,77^ In contrast, ADMSCs derived from bone pathological donors (i.e. ageing or oestrogen deficiency) preserve functional homoeostasis and therapeutic effects in locoregional bone regeneration and systemic cytotherapy for osteoporosis, indicating more resistance to a diseased microenvironment than BMMSCs.^55–61,187,188^ In addition, PDLSCs, which are recognized as the standard stem cell source for periodontal regeneration, are also more vulnerable to pro-inflammatory microenvironments than GMSCs.^186^ Therefore, further elucidation of the discrepancy among different MSC types could provide better knowledge on how to select cell sources, especially for autologous cellular therapy.

In addition to innate divergence between different MSC populations, pre-conditioning provides an efficacious approach to enhance MSC resistance to the diseased recipient microenvironment and improve MSC-based bone and dental regeneration. Genetic modification may enormously affect MSC functions via changing the expression of key genes involved in stem cell properties, which, however, may not be suitable for clinical application.^131,133^ Alternatively, epigenetic reprogramming via treatment with posttranscriptional modifiers has shown promise in achieving long-lasting resistance of MSCs to recipient microenvironmental impacts.^113,119^ For example, miR-26a overexpression rescued the impaired capacity of oestrogen-deficient mouse-derived MSCs in ectopic bone formation and in healing critical-sized calvarial bone defects.^119^ In addition, the histone methylation inhibitor DZNep enhanced MSC osteogenic potential under in vitro pathogenic conditions,^113^ which need further studies to evaluate the in vivo effects. On the other hand, pharmacological intervention with small molecule compounds has shown promise in enhancing MSC regenerative potential. Pretreating MSCs with aspirin before in vivo transplantation enhanced MSC resistance to the recipient inflammatory microenvironment, resulting in significant improvement of MSC-based ectopic bone regeneration.^34,203^ In addition, melatonin treatment promoted MSC osteogenesis abilities, resulting in enhanced local bone regeneration mediated by MSCs both in ectopic sites and in critical-sized calvarial bone defects.^204^ Notably, pre-conditioning with melatonin strengthened the therapeutic effects of systemically transplanted MSCs for OVX-induced osteoporosis with restoration of recipient bone remodelling, indicating an enhancement of their resistance to oestrogen-deficient and inflammatory microenvironments.^204^ In addition, it has been demonstrated that metformin pretreatment helped maintain MSC immunomodulation ability under high glucose conditions, leading to preservation of MSC therapeutic efficacy on osteoporosis with hyperglycaemia.^33^ Intriguingly, a low concentration of IFN-γ has been demonstrated to be essential for priming donor MSC immunomodulation property^35,205^ and to promote MSC-based bone formation.^206^ With the above approaches, the ability of MSCs to resist diseased microenvironments will be strengthened, which helps guarantee the therapeutic efficacy of these cells.

### Restoration of recipient microenvironment to benefit transplanted stem cells

Considering the role of recipient microenvironments in determining the therapeutic efficacy of transplanted stem cells, pre-normalizing the microenvironment to provide a favourable regeneration condition is another promising strategy to optimize MSC-based bone and tooth regeneration. Before local transplantation of MSCs into the injury area, the application of the anti-inflammatory agent aspirin specifically around the transplantation location inhibited the regional pro-inflammatory condition with downregulation of IFN-γ and TNF-α. As a result, MSC-mediated repair of critical-sized calvarial bone defects was promoted.^34^ In addition, for MSC-based systemic cytotherapy, the short-term application of a proteasome inhibitor, bortezomib, at arthritis onset enhanced MSC therapeutic efficacy for arthritis via inhibiting the diseased inflammatory microenvironment.^190^ Moreover, for diabetic recipients, the therapeutic effects of MSCs may be promoted by controlling the hyperglycaemic microenvironment. During systemic MSC injection, intensive infusion of insulin, which helped maintain a normal condition, guaranteed the effects of MSCs to treat osteopenia.^33^ Notably, the one-time injection of insulin at a low dose just prior to MSC infusion could enable MSCs to alleviate osteoporosis in diabetic recipients, which indicated the feasibility of building up a normoglycaemic “window” for MSC transplantation.^33^

Another approach to normalize the diseased recipient microenvironment is through the delivery of cells. For MSC-mediated ectopic bone regeneration, infusion of Tregs modulated the recipient inflammatory microenvironment, leading to improved regeneration in immunocompetent recipients.^34^ Notably, MSCs are able to modulate the recipient microenvironmental condition via cell–cell contact and paracrine secretion of a variety of cytokines and EVs.^35,36,41^ Indeed, MSC-mediated bone healing via locoregional transplantation could be enhanced by synchronized systemic MSC infusion, which inhibited recipient immunological responses and decreased inflammatory levels.^135^ Similarly, under certain conditions, multiple systemic infusions of MSCs have superior effects to one-time MSC infusion due to the modulatory effects of MSCs.^207,208^ Moreover, the two-time infusion of MSCs has been proven as effective in the treatment of diabetic osteoporosis, during which the first restored normoglycaemic conditions to create a beneficial microenvironment so that the second infused MSCs could ameliorate osteopenia.^33^

In addition to cytotherapy, establishing a favourable microenvironment is also an effective approach to promote the regenerative efficacy of MSC-based tissue engineering. The cell sheet/aggregate technique, which delivers cells with intact surface adhesion molecules and cell–cell interactions, has been recognized to establish a beneficial microenvironment for exogenously transplanted MSCs.^209^ Moreover, the bone regeneration potential of MSC aggregates could be enhanced by licochalcone A, a small molecular compound promoting ECM secretion and MSC osteogenic differentiation, resulting in facilitated healing of metaphyseal defects in oestrogen-deficient recipients.^50^ On the other hand, bioactive biomaterials with the ability to create a beneficial ambient microenvironment have been increasingly applied in tissue engineering along with bioactive molecular modification. The application of strontium-substituted calcium silicate bioactive ceramic scaffolds facilitated bone regeneration in osteoporotic recipients.^210^ In addition, akermanite bioceramics significantly promoted the healing of critical-sized calvarial defects in oestrogen-deficient mice and that calcium, magnesium and silicon-containing akermanite bioceramics enhanced the osteogenesis and angiogenesis of OVX mice-derived MSCs.^211^ Furthermore, mesoporous bioglass/silk fibrin scaffolds combined with delivery of platelet-derived growth factor B and bone morphogenetic protein-7 (BMP-7) have shown notable pro-regenerative effects on osteoporotic femoral defects.^212^ In addition, the use of calcium phosphate cement scaffolds loaded with icariin could create a beneficial niche in oestrogen-deficient conditions to increase the osteogenesis and angiogenesis of MSCs, thus promoting MSC-based osteoporotic fracture healing.^213^ Taken together, the restoration of recipient microenvironments is a feasible way to optimize MSC-based bone and dental regeneration.

## Cell-free strategies in bone and dental regeneration

As discussed above, MSCs possess a strong ability to secrete EVs containing a wide variety of proteins, lipids, and nucleic acids, which play a key role in mediating cell–cell communication. Notably, EVs have emerged as a promising alternative to whole-cell therapy with considerable potential in bone and dental regeneration.^214–216^ Exosomes, the most extensively studied EV type, have been increasingly recognized as the main components of the secretome mediating MSC therapeutic effects on osteoporosis, the systemic injection of which alleviates osteopenia as efficiently as MSCs.^39,41^ In particular, both MSCs and exosomes exert long-lasing therapeutic effects, with one-time injection resulting in restoration of bone mass for months.^39,41^ The underlying mechanism may be the modulation of recipient epigenetic states via transfer of microRNAs and proteins.^39,41^ In addition, the application of MSC-derived exosomes alone or combined with scaffolds has exhibited the potential to promote bone regeneration for fracture, defects, osteoarthritis and osteonecrosis.^40,217–221^ Furthermore, exosomes exhibit preserved therapeutic efficacy in a diseased microenvironment, as shown in the healing of critical-sized bone defects in OVX rats.^222^ The ability of exosomes to resist recipient pathogenic microenvironments has been further proven, as these molecules maintain therapeutic potential for osteoporosis under autoimmune conditions, such as SLE and systemic sclerosis.^39,41^ Given the diversity of EVs, further elucidation of the therapeutic prospects of other EVs is needed, which will promote the establishment of cell-free regeneration strategies.

Within recent years, EV-based cell-free strategies have shown encouraging therapeutic potential with superiority to single molecule drugs, whole cells, and synthetic liposome or nanoparticle formulations. EVs are easily obtained and stored sustainably and reproducibly and remain relatively stable when infused in vivo. In addition, by transferring a variety of secreted factors, EVs are able to exert synthetical effects on the recipient microenvironment with high safety. In addition, the use of engineered EVs enables the application of multiple factors with favourable biocompatibility and biostability and shows less risks for differentiation abnormalities or neoplastic transformation. Collectively, the translation of MSC-derived EVs into clinically feasible therapeutics will become a thriving strategy in bone and dental regeneration, which will stimulate an enormous amount of preclinical and clinical researches in the near future.

## Clinical trials of MSC-based bone and dental regeneration

In recent years, an increasing number of clinical trials have been conducted to assess the clinical feasibility of MSC-based regenerative therapies for bone and dental diseases (Table 1). Uncontrolled clinical trials have demonstrated that the intra-articular injection of ADMSCs is a safe and effective therapeutic approach for patients with knee osteoarthritis that significantly improves pain, function and daily living activities.^223^ For the treatment of osteonecrosis of the femoral head (ONFH), the application of bone marrow-derived mononuclear cells (BMMCs) that are highly enriched with BMMSCs leads to a notable alleviation of clinical symptoms, improved hip function and delayed collapse, thus constituting a safe, efficacious and minimally invasive treatment approach, especially for patients at early stages of ONFH.^224–227^ In addition, in one clinical study, patients with calvarial defects underwent a cranioplasty procedure using a combination of ADMSCs and beta-tricalcium phosphate granules, which promotes reconstruction of large cranial defects.^228^ In addition, the clinical relevance of MSCs in dental regeneration has also been assessed. In a randomized controlled clinical study conducted by our group, SHED aggregates could regenerate complete pulp tissues in patients with pulp necrosis, which is equipped with blood vessels and nerves.^71^ More importantly, after SHED implantation, the recipient immature permanent teeth showed increased root length and decreased apical foramina width, indicating that the regenerated pulp acted as normal pulp to maintain continued root development.^71^ For patients with severe mandibular ridge resorption, researchers inserted BMMSCs into the resorbed alveolar ridge together with biphasic calcium phosphate granules as scaffolds, which induced significant new bone formation that was adequate for dental implant installation.^229^ Furthermore, a single-centre, randomized trial was conducted to assess the efficacy of PDLSCs in combination with bovine-derived bone mineral materials for the treatment of periodontal intrabony defects. No statistically significant differences were detected between the experimental group and the control group, which needs to be further validated by multicentre randomized controlled studies.^230^

**Table 1:** Clinical trials of stem cell-based therapies for dental regeneration

Indication	Cell source	Phase, patients	Results/status	Clinical trial
Endodontic inflammation	SHED	N/A, *n* = 80	Unknown status	NCT01814436
	DPSCs	N/A, *n* = 30	Completed (no results posted)	NCT02842515
	Stem and progenitor cells	Phase 3, *n* =29	Active, not recruiting	NCT02437708
Periapical periodontitis	Umbilical cord-derived MSCs	N/A, *n* = 38	Completed (no results posted)	NCT03102879
Periodontitis	DPSCs	N/A, *n* = 29	Completed (no results posted)	NCT03386877
		Phase 1/2, *n* = 40	Unknown status	NCT02523651
	PDLSCs	Phase 1, *n* = 35	Unknown status	NCT01357785
		Phase 1/2, *n* = 80	Unknown status	NCT01082822
	BMMSCs	Phase 1/2, *n* = 30	Completed (no results posted)	NCT02449005
	GMSCs	Phase 1/2, *n* = 30	Recruiting	NCT03137979
	MSCs	Phase 1/2, *n* = 10	Completed (no results posted)	NCT00221130
Alveolar bone loss	DPSCs	Phase 1, *n* = 10	Enrolling by invitation	NCT02731586
	Buccal fat pad derived stem	Phase 1, *n* = 20	Unknown status	NCT02745379
	cells	Phase 1, *n* = 20	Unknown status	NCT02745366
	Oral mucosa MSCs	Phase 1/2, *n* = 12	Unknown status	NCT02209311
	GMSCs	N/A, *n* = 20	Completed (no results posted)	NCT03638154
Cleft lip and palate	DPSCs	Phase 3, *n* = 62	Not yet recruiting	NCT03766217
		NA, *n* = 5	Completed (satisfactory bone healing)	NCT01932164
Cleft of alveolar ridge	Buccal fat pad derived stem cells	Phase 1, *n* = 10	Completed (no results posted)	NCT02859025
Jaw bone atrophy	MSCs	Phase 1, *n* = 13	Enrolling by invitation	NCT02751125

However, despite the promising results of these studies, there are still many obstacles limiting the use of MSCs in clinical bone and dental regeneration. Many of the completed clinical trials registered in ClinicalTrials.gov have not provided results, which may restrain the clinical transformation of MSC-based regenerative therapies. In addition, the development of internationally recognized, standardized guidelines on cell selection, expansion, storage and shipping are needed to provide clinically applicable cell sources. Another aspect that needs to be addressed is the lack of a standardized procedure for cytotherapy or the application of MSC-based tissue engineering products. More importantly, the fulfilment of the function of transplanted cells requires technological advances that optimize the retention, viability, homing, differentiation ability and modulatory capacity of MSCs in vivo.

## Conclusion

Over the past several years, MSC-based regeneration strategies have shown great promise for healing bone and dental loss and defects, both via endogenous restoration and exogenous transplantation. Notably, the therapeutic efficacy of MSC-mediated regeneration is under tight control of the microenvironment, which not only regulates resident MSCs under both physical and pathological conditions but also modulates transplanted MSCs in cytotherapy and tissue engineering. As a result, achieving MSC-based bone and dental regeneration in diseased microenvironments remains a major challenge. Accordingly, microenvironment-targeting therapeutic strategies that may promote the optimization of MSC-based bone and dental healing in diseased microenvironments have been established. In this regard, several tactics have demonstrated enormous potential, including improvement of the endogenous microenvironment to revitalize innate MSCs, modification via pharmacological or epigenetic approaches to enhance exogenous MSC resistance, and restoration of the recipient microenvironment to benefit transplanted MSCs. Notably, EVs/exosomes have emerged as attractive alternatives to MSCs in both cytotherapy and tissue engineering with pro-regenerative potential and microenvironment modulatory abilities (Fig. 4).

While much progress has been achieved, several issues remain to be explored. First, further studies regarding the microenvironmental modulation of MSC-based tissue regeneration and underlying molecular mechanisms are needed to pinpoint the specific contributions of the microenvironment to MSC-based therapies and identify key molecules and signalling pathways involved. Second, the application of novel techniques to improve MSC-based bone and dental regeneration, such as modifying biomimicking materials via nanotechnology to establish a bionic microenvironment^231–233^ and strengthening MSC recruitment via an aptamer-targeting technique to promote oriented transplantation, is needed.^234,235^ Third, given the control of the microenvironment over MSCs, it is advisable to analyse the recipient microenvironment status and accordingly formulate therapeutic time points prior to MSC transplantation to strengthen the efficacy of infused MSCs. Last but not least, in addition to prolonging the survival of transplanted MSCs, recent studies have revealed that the apoptosis of MSCs may constitute a critical mechanism underlying their therapeutic efficacy in certain disorders,^188,236^ which may provide novel insights into MSC-based regenerative therapies. In summary, understanding the effects of microenvironmental modulation on MSCs will shed more light on the pathogenesis and therapeutics of bone and tooth, which will promote the establishment of optimized MSC-based strategies for bone and dental regeneration.
